# Data on a simple method for producing a solution that contains a high partial pressure of oxygen and a low partial pressure of carbon dioxide

**DOI:** 10.1016/j.dib.2018.02.079

**Published:** 2018-03-08

**Authors:** Yoshihiro Tange, Shigenori Yoshitake

**Affiliations:** Department of Medical Engineering, Kyushu University of Health and Welfare, Japan

## Abstract

The data presented here shows a simple method for producing a solution that contains a high partial pressure of oxygen (pO_2_) and a low partial pressure of carbon dioxide (pCO_2_). This novel solution was created by simply injecting oxygen gas into conventional supplemental bicarbonate fluid for renal replacement therapy. We compared the gas profiles of the novel solution and the conventional fluid in vitro. There was a significant increase in pO_2_ and pH, and a significant decrease in pCO_2_ in the experimental solution, in each of which an additional volume of oxygen was injected. The method shown here is capable of facilitating an increase of pO_2_ and decrease of pCO_2_ by using a closed fluid bag without any special devices.

**Specifications Table**

**Table:** 

Subject area	*Medicine*
More specific subject area	*Biotechnology*
Type of data	*Table, figure*
How data was acquired	*Gas profiles measured by EG6+ cartridge (Abbott Japan Co., Ltd, Osaka, Japan) with an i-STAT system (300F, Abbott, Japan)*
Data format	*Analysed data*
Experimental factors	*Simply injected oxygen gas into supplemental fluid bag.*
Experimental features	*Samples were prepared by injecting oxygen gas via a syringe into conventional bicarbonate supplemental fluid (sublood BSG, Fuso, Osaka, Japan). The volumes of oxygen injected into the fluid were zero for the control solution, and 500, 1000, and 1400 mL into 2020 mL of the supplemental fluid for the experimental solution.*
Data source location	*Nobeoka City, Miyazaki, Japan*
Data accessibility	*All data are included in this document.*

**Value of the data**

• A solution with high pO_2_ and low pCO_2_ was obtained using a simple method.
• This method only requires oxygen gas.
• This method did not require any special devices, unlike those previously reported.

## 1 Data

Intravenous fluid with a high partial pressure of oxygen (pO_*2*_) was shown to improve hypoxia in several animal models [1], [2], [3]. Further studies demonstrated that fluids containing high amounts of dissolved oxygen achieved supersaturated oxygen levels in the bloodstream, but special devices were needed to create these fluids [4], [5]. If such fluids were simpler to create, they could be used easily at the bedside. The data presented here shows a simple method for producing a solution that contains a high pO_2_ and a low pCO_2_, using the supplemental fluid.

## 2 Experimental design, materials and methods

### 2.1 Materials

Samples were prepared using a conventional bicarbonate supplemental fluid (sublood BSG, Fuso, Osaka, Japan); the air was removed using a syringe (Nipro, Osaka, Japan). The composition of supplemental fluid is shown in Table 1.

**Table 1:** Detail components in the supplemental fluid.

Bags	Components
A solution	NaCl, KCl, NaHCO_3_
B solution	NaCl, KCl, CaCl_2_·2H_2_O, MgCl_2_·6H_2_O, CH_3_COONa, C_6_H_12_O_6_

### 2.2 Methods

**Fig. 1:**
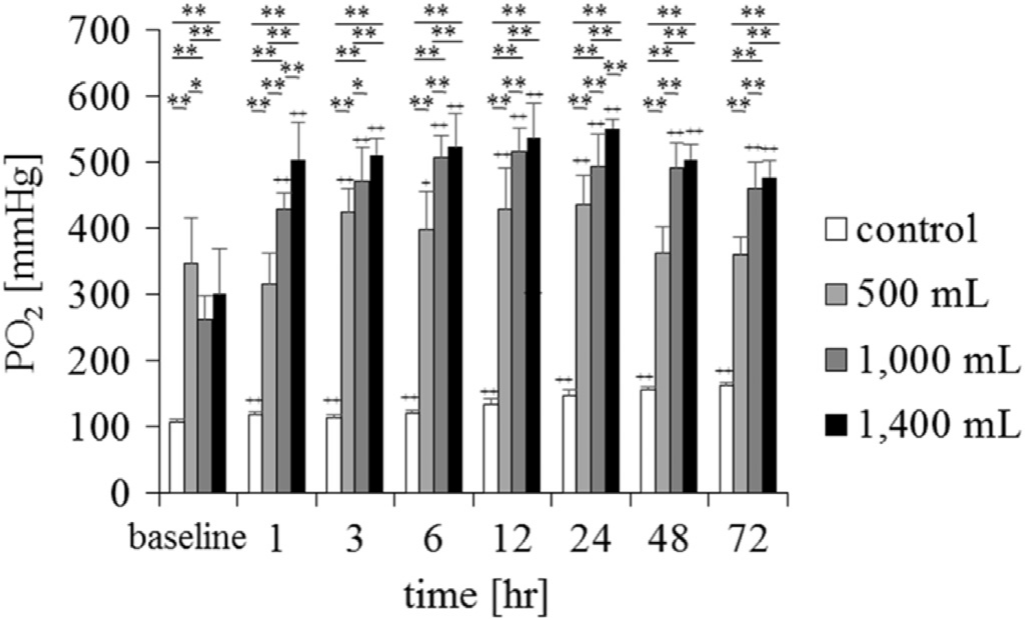
Changes in pO_2_ in the conventional supplemental fluid and a supersaturated oxygen solution (*n* = 6, mean ± standard deviation). The control was a conventional supplemental solution, and the experimental solution was created by injecting 500, 1000, and 1400 mL of oxygen into 2020 mL of the supplemental solution. **P < 0.01, *P < 0.05, comparison between groups; ++*P* < 0.01, + *P* < 0.05 vs baseline. There was a significant increase in pO_2_ in the control group after 1 h vs at the baseline. In the experimental groups, there was a significant increase in pO_2_ until 72 h after the injection of 500 mL, 1000 mL, and 1400 mL of oxygen. The time course of the experimental and control solutions was compared using repeated-measures analysis of variance, and the groups were compared with Bonferroni correction, as appropriate.

**Fig. 2:**
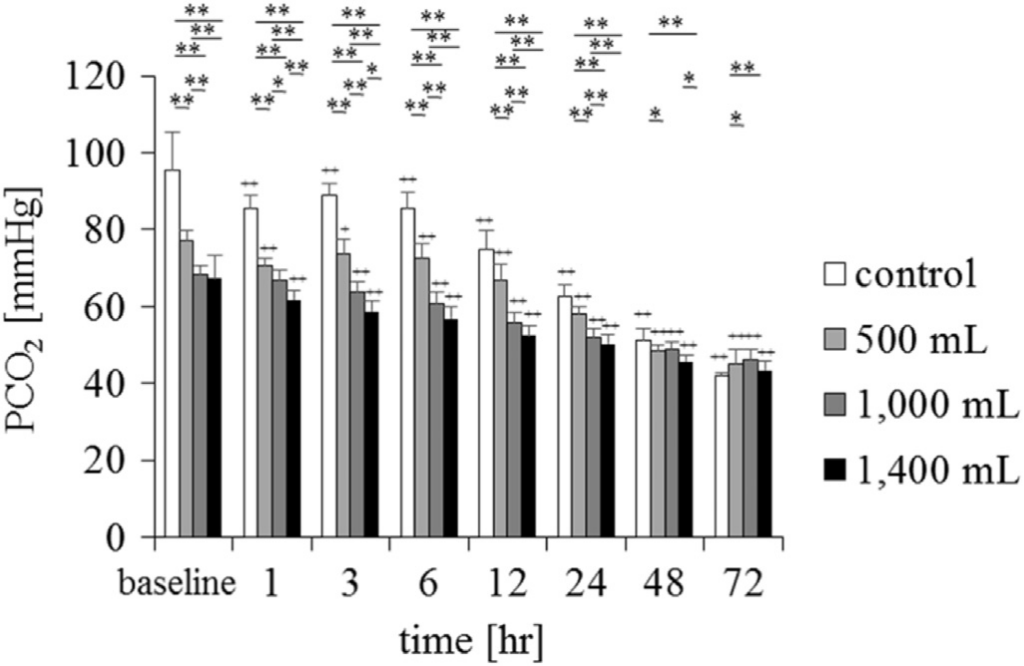
Changes in pCO_2_ in the conventional supplemental fluid and a supersaturated oxygen solution (*n* = 6, mean ± standard deviation). The control was a conventional supplemental solution, and the experimental solution was created by injecting 500, 1000, and 1400 mL of oxygen into 2020 mL of supplemental solution. **P < 0.01, *P < 0.05, comparison between groups; ++*P* < 0.01, +*P* < 0.05 vs baseline. There was a significant decrease in pCO_2_ after 1 h when compared to the baseline value. The time course of the experimental and control solutions was compared using repeated-measures analysis of variance, and the groups were compared with Bonferroni correction, as appropriate.

**Fig. 3:**
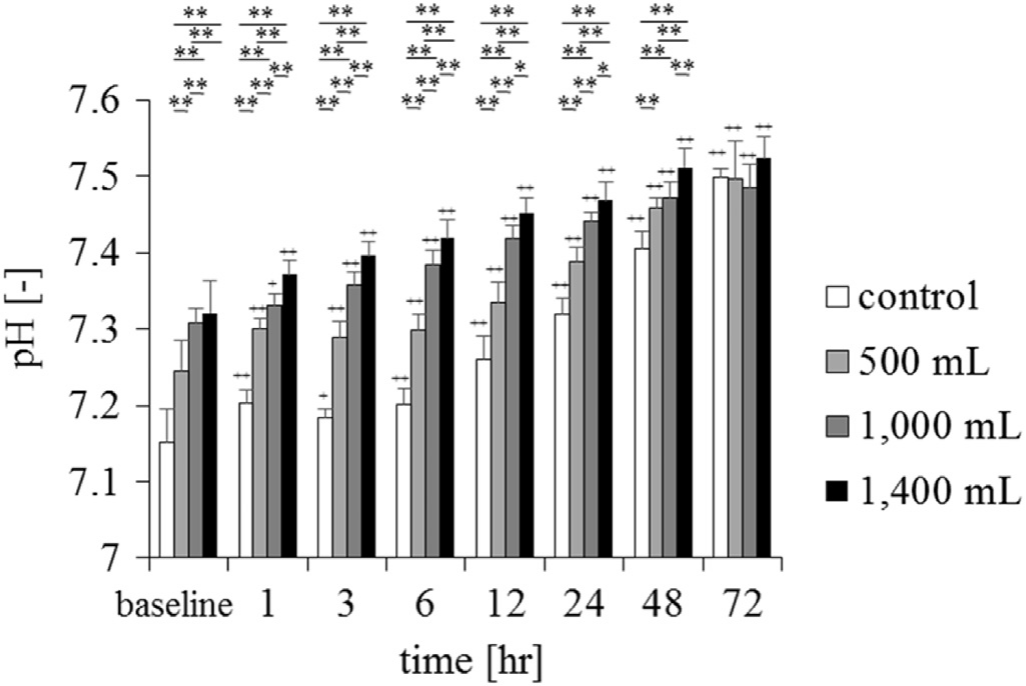
Changes in pH in the conventional supplemental fluid and a supersaturated oxygen solution (*n* = 6, mean ± standard deviation). The control was a conventional supplemental solution, and the experimental solution was created by injecting 500, 1000, and 1400 mL of oxygen into 2020 mL of supplemental solution. **P < 0.01, *P < 0.05, comparisons between groups; +*P* < 0.05, ++*P* < 0.01 vs baseline. There was a significant increase in pH after 1 h when compared to the baseline value, which was constant until 48 h in all the oxygen-injected groups. The time course of the experimental and control solutions was compared using repeated-measures analysis of variance, and the groups were compared with Bonferroni correction, as appropriate.

The samples were injected with oxygen gas via a syringe connected to an oxygen piping line. The volumes of oxygen injected into the fluid were zero for the control solution, and 500, 1000, and 1400 mL into 2020 mL of supplemental fluid for the experimental solution. To determine the gas profile in the supplemental fluid, we sampled the fluid in bags containing dissolved oxygen after shaking them for one minute. We determined the pO_2_, pCO_2_, and pH values using an EG6+ cartridge (Abbott Japan Co., Ltd, Osaka, Japan) with an i-STAT system (300F, Abbott, Japan). Samples were analysed immediately after the injection of oxygen for obtaining the baseline data, and for up to 72 h subsequently (*n* = 6). The room temperature was set at 24 ± 0.5 °C. Fig. 1, Fig. 2, Fig. 3 show the changes in the gas profiles of the solutions. The values for the supplemental fluid immediately after the injection of 500, 1000, and 1400 mL of oxygen were set as the baseline values.
