# Supplementary material for: Data on a simple method for producing a solution that contains a high partial pressure of oxygen and a low partial pressure of carbon dioxide
Source: Data Brief. 2018 Mar 8;18:176–9. doi: 10.1016/j.dib.2018.02.079 (PMC5996131; doi:10.1016/j.dib.2018.02.079)
Supplement: Supplementary file 1 — Transparency document [file mmc1.docx]

**Conflict of interests**

None declared.
